# *CDKN2A* methylation in esophageal cancer: a meta-analysis

**DOI:** 10.18632/oncotarget.18412

**Published:** 2017-06-08

**Authors:** Chongchang Zhou, Jinyun Li, Qun Li

**Affiliations:** ^1^ Department of Otorhinolaryngology Head and Neck Surgery, Lihuili Hospital of Ningbo University, Ningbo 315040, Zhejiang, China; ^2^ Department of Medical Oncology, Affiliated Hospital of Ningbo University, Ningbo 315000, Zhejiang, China

**Keywords:** *CDKN2A*, methylation, diagnosis, esophageal cancer, carcinogenesis

## Abstract

*CDKN2A* is a tumor suppressor gene and is frequently inactivated in human cancers by hypermethylation of its promoter. However, the role and diagnostic value of *CDKN2A* methylation in esophageal cancer (EC) remains controversial. Therefore, we performed a meta-analysis, including data from 42 articles (2656 ECs, 612 precancerous lesions, and 2367 controls). A significant increase in the frequency of *CDKN2A* methylation was identified during EC carcinogenesis: cancer vs. controls, odds ratio (OR) = 12.60 (95 % CI, 8.90–17.85); cancer vs. precancerous lesions, OR = 2.89 (95% CI, 2.20–3.79); and precancerous lesions vs. controls, OR = 7.38, 95% (CI, 4.31–12.66). *CDKN2A* promoter methylation was associated with EC tumor grade (OR = 1.79; 95% CI, 1.20–2.67) and clinical stage (OR = 2.56; 95% CI, 1.33–4.92). Additionally, the sensitivity, specificity, and area under the summary receiver operating characteristic curve (AUC) for diagnosis of EC based on *CDKN2A* methylation were 0.52 (95% CI, 0.44–0.59), 0.96 (95% CI, 0.93–0.98), and 0.83 (95% CI, 0.79–0.86), respectively. AUCs for blood and tissue sample subgroups were 0.90 and 0.82, respectively. Our findings indicate that *CDKN2A* methylation has a vital role in EC tumorigenesis and could be a biomarker for early diagnosis of EC.

## INTRODUCTION

Esophageal cancer (EC) is the eighth most common and the sixth most deadly cancer worldwide [1]. Esophageal adenocarcinoma (EAC) and esophageal squamous cell carcinoma (ESCC) are two major histologic subtypes of EC. EAC is more common in western Europe and north America, whereas ESCC is more prevalent in south-eastern and central Asia, particularly China and Japan [2]. In China alone, 477,900 newly diagnosed EC cases and 375,000 deaths from EC were projected to occur in 2015 [3]. Despite recent advances in combination therapy strategies, including surgery, chemotherapy, and radiotherapy, the prognosis for EC patients remains unsatisfactory, especially for advanced-stage patients, and the 5-year overall survival rate remains at less than 20% [4]. The rapidly increasing incidence, demanding treatment, and poor outcomes of EC highlight the need for effective potential biomarkers for early diagnosis, prognosis prediction, and novel therapeutic targets.

The etiology and pathogenesis of EC involve complicated interactions between epigenetic, genetic, and environmental factors [5–7]. DNA methylation is a common form of epigenetic modification, which has a crucial role in human malignancies, such as breast [8], lung [9], and gastric [10] cancers. Abnormal methylation in the promoter region of tumor suppressor genes is one of the most common mechanisms of modification, and results in target gene transcriptional silencing. Moreover, with the introduction of precise and convenient methods of detection, DNA methylation has become a credible potential biomarker for early detection and diagnosis of cancer [11].

The cyclin-dependent kinase inhibitor 2A (*CDKN2A*) gene on chromosome 9p21 is a classical tumor suppressor gene [12]. It is responsible for inhibiting various cyclin-dependent kinases and plays an important role in cell cycle regulation by decelerating cell cycle progression at the G1/S phase [13, 14]. Hypermethylation of the *CDKN2A* gene promoter region, resulting in its inactivation, has been reported in several types of malignancy, including lung [15], head and neck [16], hepatocellular [17], breast [18], and esophageal [19] cancers. Although they have been frequently investigated, the association between *CDKN2A* promoter methylation and EC and the role of this modification in EC carcinogenesis, remain controversial. For example, one study reported that the methylation rate at the *CDKN2A* promoter was similar in EC patients and healthy controls [20]; however, another investigation identified a differential frequency of *CDKN2A* promoter methylation between these two groups [21]. In addition, the value for EC diagnosis of testing for *CDKN2A* methylation, particularly using blood samples, has been less intensely investigated.

Here, we performed a meta-analysis to generate a quantitative estimate of the association of *CDKN2A* methylation with EC risk and its role in EC carcinogenesis. Furthermore, we assessed whether there were associations between *CDKN2A* promoter methylation and the clinical characteristics of EC patients. In addition, we comprehensively evaluated the diagnostic utility of *CDKN2A* methylation for EC, to assess the future potential applicability of *CDKN2A* methylation testing for the prevention, diagnosis, and therapy of EC.

## RESULTS

### Study characteristics

**Figure 1:**
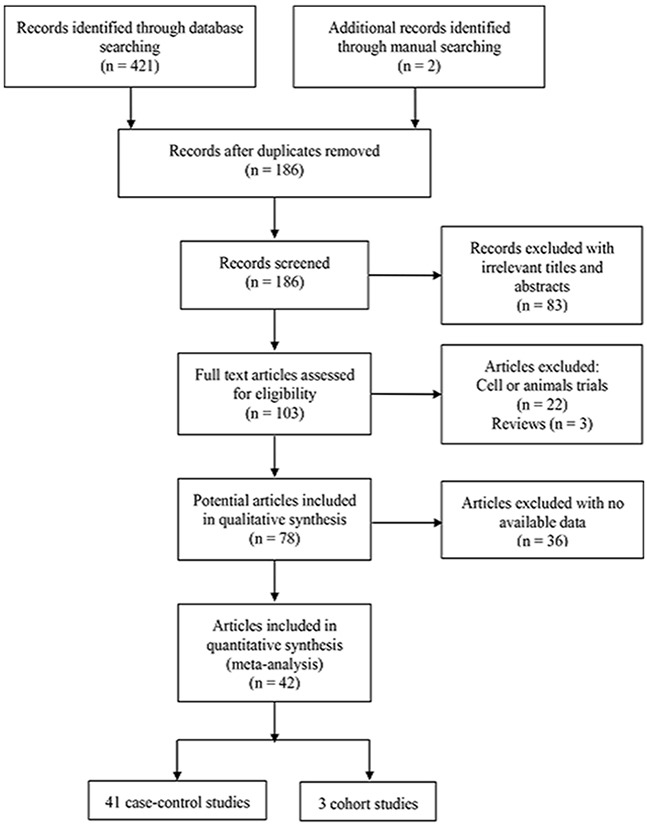
Flow diagram of the study search strategy for this meta-analysis

The flow chart of the selection process for inclusion of studies in our analysis is presented in Figure 1. A total of 423 articles were initially identified, with 421 identified by database, and two by manual searching. After a careful initial review of the titles and abstracts, 237 duplicate and 83 irrelevant articles were excluded. Next, we reviewed the full text of the remaining articles. Among these, 61 articles were excluded, since 22 were focused on cell lines or animals trials, three were reviews, and 36 lacked methylation data. Finally, 42 articles including 44 studies (41 case-control and 3 cohort studies) were included in the meta-analysis. The basic characteristics of all eligible studies are presented in Table 1.

**Table 1:** The main characteristics of studies included in the analysis of the association of *CDKN2A* promoter methylation with esophageal cancer

First author	Year	Country	Ethnicity	Method	Histology	Type	Carcinoma		Normal			Pre-carcinoma	
							M+	Total	M+	Total	Source	M+	Total
Wang	2011	China	Asian	MSP	ESCC	Tissue	6	13	0	12	H	14	64
Song	2009	China	Asian	MSP	ESCC	Tissue	41	130	18	260	A	Na	Na
Song	2007	China	Asian	MSP	ESCC	Tissue	65	140	31	280	A	Na	Na
Yan	2003	China	Asian	MSP	ESCC	Tissue	5	34	0	6	H	Na	Na
Guo	2008	China	Asian	MSP	ESCC	Tissue	35	51	1	10	H	18	44
Yu	2006	China	Asian	MSP	ESCC	Tissue	33	45	26	45	A	Na	Na
Guo	2006	China	Asian	MSP	ESCC	Blood	14	51	0	10	H	0	44
Zhao	2011	China	Asian	MSP	ESCC	Tissue	31	71	10	71	A	Na	Na
									2	71	H	Na	Na
Wang	2012	China	Asian	qMSP	ESCC	Tissue	66	76	3	76	A	Na	Na
Wang	2012	China	Asian	qMSP	ESCC	Blood	54	76	2	60	H	Na	Na
Yao	2005	China	Asian	nMSP	ESCC	Blood	34	56	0	22	H	Na	Na
Wang	1997	USA	Caucasian	MSP	EAC	Tissue	5	11	0	21	A	3	10
Kempste	2000	Australia	Caucasian	MSP	ESCC	Tissue	3	7	0	7	A	Na	Na
					EAC	Tissue	6	9	0	9	A	Na	Na
Bian	2002	Switzerland	Caucasian	MS-SSCA	EAC	Tissue	18	22	0	10	H	10	33
Nie	2002	China	Asian	MSP	ESCC	Tissue	7	21	0	25	A	5	13
Sarbia	2004	Germany	Caucasian	qMSP	EAC	Tissue	27	50	0	50	A	Na	Na
Zhang	2004	China	Asian	MSP	ESCC	Tissue	5	34	0	6	H	Na	Na
Schulmann	2005	USA	Caucasian	qMSP	EAC	Tissue	34	76	2	64	A	14	93
Abbaszadegan	2005	Iran	Caucasian	MSP	ESCC	Tissue	22	30	0	30	H	Na	Na
Abbaszadegan	2005	Iran	Caucasian	MSP	ESCC	Blood	13	30	0	30	H	Na	Na
Roth	2006	China	Asian	MSP	ESCC	Tissue	5	13	0	11	H	3	15
Clement	2006	Switzerland	Caucasian	MS-SSCA	EAC	Tissue	13	27	0	16	H	Na	Na
Guo	2006	China	Asian	MSP	ESCC	Tissue	36	69	0	17	H	20	60
Ishii	2007	Japan	Asian	COBRA	ESCC	Tissue	22	56	18	56	A	8	21
						Tissue			0	42	H	Na	Na
Wang	2008	China	Asian	MSP	ESCC	Tissue	110	125	46	125	A	Na	Na
						Tissue			0	10	H	Na	Na
Salam	2009	India	African	MSP	ESCC	Tissue	36	69	2	69	A	Na	Na
Wang	2009	USA	Caucasian	MSP	ESCC	Tissue	22	41	0	17	H	33	92
Ganji	2010	Italy	Caucasian	MSP	ESCC	Tissue	12	44	0	19	H	Na	Na
Taghavi	2010	Iran	Caucasian	MSP	ESCC	Tissue	31	50	2	50	A	Na	Na
Lu	2011	China	Asian	MSP	ESCC	Tissue	106	120	46	120	A	Na	Na
Ikoma	2007	Japan	Asian	MSP	ESCC	Blood	6	44	0	12	H	Na	Na
Xing	1999	China	Asian	MSRE	ESCC	Tissue	17	34	3	34	A	Na	Na
Brock	2003	Japan	Asian	MSP	EAC	Tissue	16	41	10	41	A	Na	Na
Vieth	2004	Germany	Caucasian	MSP	EAC	Tissue	8	15	0	15	A	17	55
Hardie	2005	UK	Caucasian	MSP	EAC	Tissue	18	21	9	21	H	14	18
Li	2011	China	Asian	MSP	ESCC	Tissue	21	47	10	47	A	Na	Na
Ling	2010	China	Asian	MSP	ESCC	Tissue	25	50	1	50	H	10	50
Chen	2012	China	Asian	MSP	ESCC	Tissue	210	257	75	257	A	Na	Na
Talukdar	2013	India	African	MSP	ESCC	Tissue	42	112	2	30	A	Na	Na
Liao	2009	China	Asian	MSP	ESCC	Tissue	53	105	8	105	A	Na	Na
Hoshimoto	2015	USA	Caucasian	qMSP	ESCC	Tissue	27	114	0	28	A	Na	Na
Das	2014	India	African	MSP	ESCC	Blood	81	100	Na	Na	Na	Na	Na
Ito	2007	Japan	Asian	MSP	ESCC	Tissue	29	38	Na	Na	Na	Na	Na
Hibi	2001	Japan	Asian	MSP	ESCC	Blood	7	31	Na	Na	Na	Na	Na

M+, positive for *CDKN2A* methylation; MSP, methylation specific polymerase chain reaction; qMSP, quantitative methylation specific polymerase chain reaction; MSRE, methylation sensitive restriction endonuclease; nMSP, nested methylation specific polymerase chain reaction; MS-SSCA, methylation-sensitive-single-strand conformation analysis; COBRA, combined bisulfite restriction analysis; A, autologous (control sample from the same patients); H, heterogeneous (control samples from other individuals); ESCC, esophageal squamous cell carcinoma; EAC, esophageal adenocarcinoma; Na, not available.

### Comparison of the frequency of methylation of the *CDKN2A* promoter in EC cases and healthy controls

**Figure 2:**
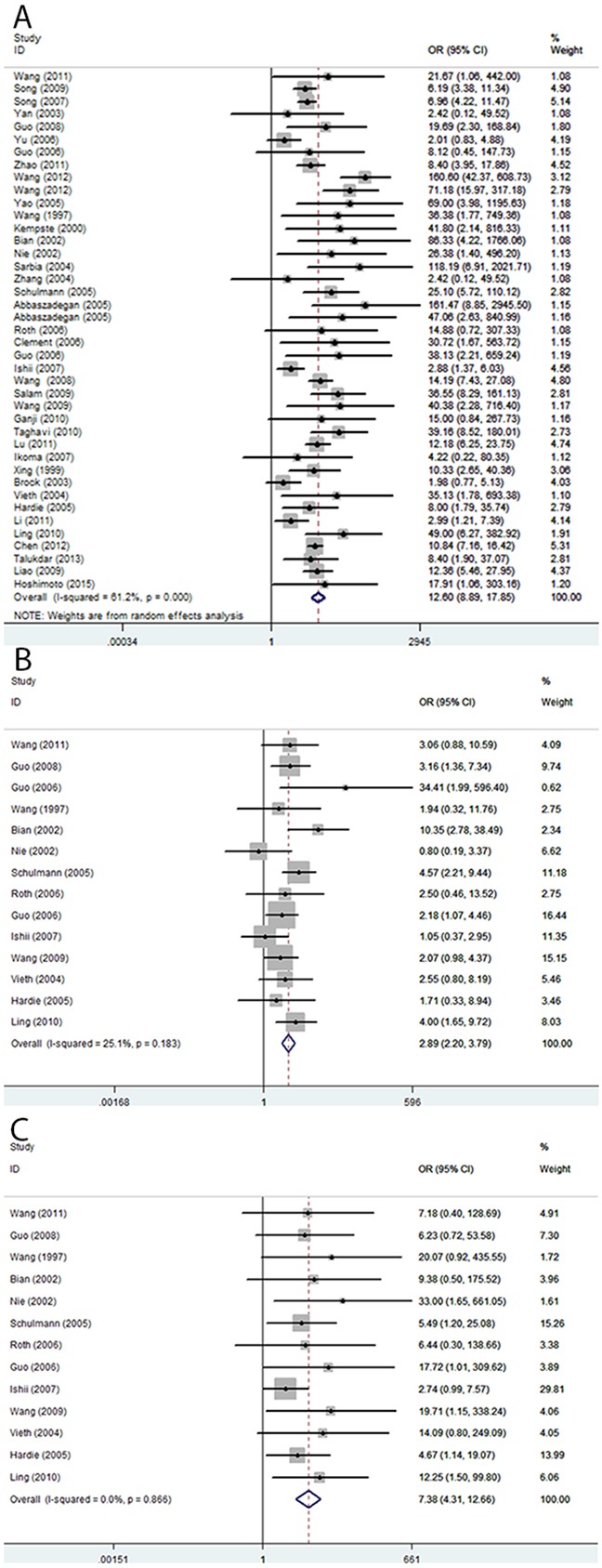
Pooled forest plot of *CDKN2A* methylation status during the carcinogenesis of esophageal cancer **(A)** Cancer vs. controls: OR = 12.60; 95 % CI, 8.90–17.85. **(B)** Cancer vs. precancerous lesions: OR = 2.89; 95% CI, 2.20–3.79. **(C)** Precancerous lesions vs. control: OR = 7.38; 95% CI, 4.31–12.66.

**Figure 3:**
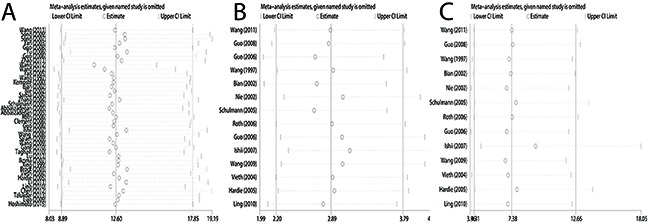
Sensitivity analysis of pooled odds ratios for *CDKN2A* methylation during the carcinogenesis of esophageal cancer **(A)** Cancer vs. controls. **(B)** Cancer vs. precancerous lesions. **(C)** Precancerous lesions vs. control.

**Figure 4:**
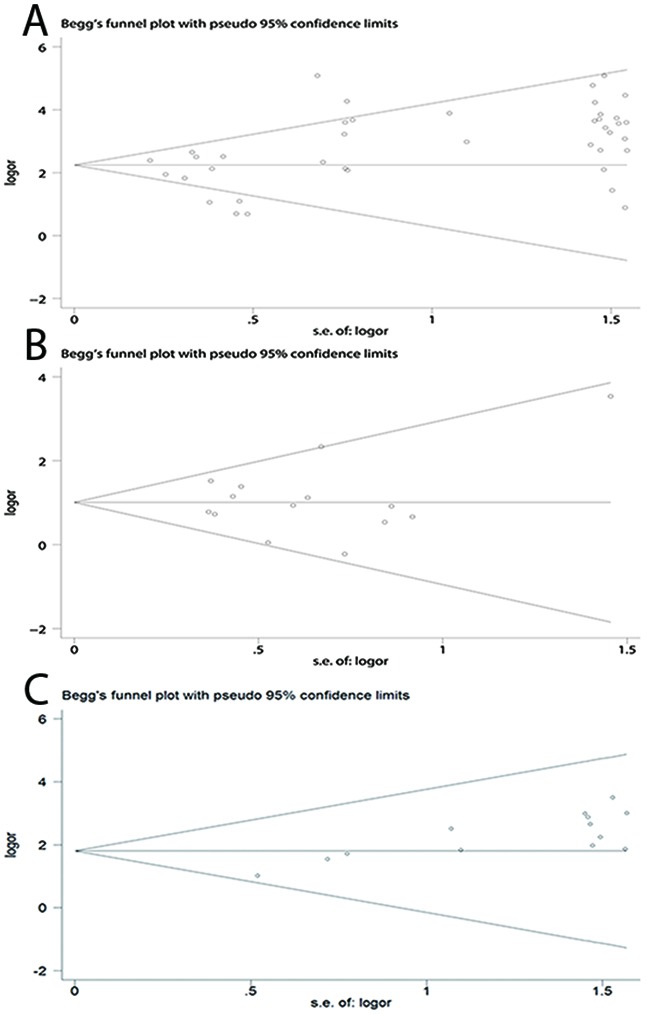
Begg's funnel plots of publication bias for *CDKN2A* methylation during the carcinogenesis of esophageal cancer **(A)** Cancer vs. controls. **(B)** Cancer vs. precancerous lesions. **(C)** Precancerous lesions vs. control.

A total of 41 case-control studies, including 2487 EC cases and 2367 healthy controls, were included in the current meta-analysis. Our results indicated that the frequency of methylation of the *CDKN2A* promoter was significantly higher in EC cases than in healthy controls (odds ratio (OR) = 12.60, 95 % CI = 8.90–17.85; Figure 2A). Meta-regression and subgroup analysis were applied to investigate potential sources of the substantial heterogeneity that was detected among the studies. Although, meta-regression analysis did not identify any obvious source of heterogeneity (Table 2), decreased heterogeneity was observed in several of the subgroup analyses (conducted according to ethnicity, sample source, control type, and detection method; Table 3), suggesting that the heterogeneity may derive from multiple sources. Additionally, all subgroups showed that *CDKN2A* hypermethylation was significantly associated with EC. Moreover, the subgroup analysis showed that the OR of the EAC subgroup (OR = 19.03) was greater than that of the ESCC subgroup (OR = 12.00), and the OR of the Caucasian subgroup (OR = 32.67) was greater than that of the Asian (OR = 9.37) and African subgroups (OR = 17.59), as well as the OR of the blood sample subgroup (OR = 34.98) was greater than that of the tissue sample subgroup (OR = 11.60). To test the robustness of our results, a sensitivity analysis to determine the influence of single studies on overall pooled ORs was performed, and the results supported the stability and credibility of our data (Figure 3A). Application of Begg's funnel plot analysis indicated no significant publication bias among the studies (P = 0.47; Figure 4A).

**Table 2:** Meta regression analyses of *CDKN2A* promoter methylation in esophageal cancer

Heterogeneity source	Coefficient	95% CI		*P*
		Lower	Upper	
Publication year	0.031	−0.097	0.159	0.625
Sample size	0.003	−0.005	0.011	0.449
Ethnicity				
Asian	−0.831	−2.42	0.759	0.295
Caucasian	0.672	−1.197	2.54	0.469
Detection method				
MSP	−0.883	−1.79	0.024	0.056
Sample source				
Tissue	−0.248	−1.901	1.405	0.762
Histology				
ESCC	0.721	−0.581	2.022	0.268
Control type				
Heterogeneous	0.64	−0.426	1.706	0.23

**Table 3:** Subgroup analyses of *CDKN2A* promoter methylation in esophageal cancer

Subgroup	Case		Control		Pooled OR (95% CI)	Heterogeneity	
	M	U	M	U		*I^2^*(%)	*P*
Race							
African	259	288	13	374	17.59 (6.20–49.94)	47	0.17
Caucasian	1023	736	310	1571	32.67 (17.61–60.62)	0	0.93
Asian	78	103	4	95	9.37 (6.28–13.99)	68.7	< 0.01
Sample source							
Tissue	1239	991	325	1908	11.60 (8.14–16.54)	61.8	< 0.01
Blood	121	136	2	132	34.98 (12.01–101.85)	2	0.4
Histology							
ESCC	1215	1000	306	1814	12.00 (8.35–17.26)	60.9	< 0.01
EAC	145	127	21	226	19.03 (6.03–60.11)	62.4	< 0.01
Control type							
Autologous	1017	764	312	1573	9.75 (6.33–15.04)	75.5	< 0.01
Heterogeneous	506	452	15	467	28.57 (16.96–48.12)	0	0.69
Methods							
MSP	1048	852	299	1610	9.67 (5.46–27.95)	47	< 0.01
Not MSP	312	275	28	430	31.50 (9.66–102.73)	78.7	< 0.01
Sample size							
< 60	449	498	80	644	5.64 (3.078–10.333)	57.7	< 0.01
≥ 60	911	629	247	1396	10.948 (1.247–96.116)	62.2	< 0.01
Publication year							
< 2010	729	728	174	1302	10.74 (7.00–16.49)	54.9	< 0.01
≥ 2010	631	399	153	738	16.83 (9.17–30.89)	68.9	< 0.01

M, methylated; U, unmethylated.

### Comparison of the frequency of methylation of the *CDKN2A* promoter in EC and precancerous lesions

There were 14 studies involving 510 ECs and 612 precancerous lesions included in this analysis. In the absence of heterogeneity (I^2^ = 25%, P = 0.183), a fixed-effects model was applied to evaluate the association of between methylation of *CDKN2A* in EC and precancerous lesions. We observed that the frequency of *CDKN2A* methylation was significantly higher in EC than precancerous lesion samples (OR = 2.89; 95% CI, 2.20–3.79; Figure 2B). The results of a sensitivity analysis indicated the authenticity of our results (Figure 3B) and a Begg's test for publication bias was not statistically significant (P = 0.74; Figure 4B)

### Comparison of the frequency of methylation of the *CDKN2A* promoter between precancerous esophageal lesions and healthy controls

The analysis of the association between methylated *CDKN2A* and esophageal precancerous lesions included 612 precancerosis samples and 381 healthy controls from 14 studies. As demonstrated in Figure 2C, the methylation frequency of *CDKN2A* was significantly elevated in precancerous lesions compared with controls (OR = 7.38; 95% CI, 4.31–12.66). Sensitivity analysis confirmed the robustness of our results (Figure 3C) and a Begg's test revealed no publication bias (P > 0.05, Figure 4C).

### Association of methylation of the *CDKN2A* promoter with clinicopathological features of EC

A total of 1313 ECs from 16 studies were used to evaluate the relationships between *CDKN2A* methylation and clinicopathological features, including age, gender, smoking behavior, alcohol consumption, tumor location, tumor diameter, differentiation grade, tumor stage, clinical stage, and lymph node metastasis (Table 4). Our findings demonstrated that *CDKN2A* methylation was significantly associated with differentiation grade (poor vs. well and moderate: OR = 1.79; 95% CI, 1.20–2.67; P < 0.01) and clinical stage (advanced vs. early: OR = 2.56; 95% CI, 1.33–4.92; P < 0.01). However, there were no associations between other clinicopathological characteristics and *CDKN2A* promoter methylation in EC.

**Table 4:** The association of *CDKN2A* promoter methylation with clinicopathological features of esophageal cancer patients

Characteristic	No.	Case type/control type	Cases/controls	OR (95% CI)	*P*	Heterogeneity	
***I*^2^ (%)**	***P***
Age	8	Older/younger	259/230	0.98 (0.65–1.47)	0.91	0	0.45
Gender	12	Male/female	568/259	0.97 (0.68–1.38)	0.84	0	0.83
Smoking behavior	8	Yes/no	409/354	1.10 (0.54–2.28)	0.79	71.2	0
Alcohol consumption	5	Yes/no	282/322	0.92 (0.47–1.82)	0.82	59.1	0.04
Differentiation grade	11	Poor/well and moderate	144/614	1.79 (1.20–2.67)	0	25.2	0.2
T stage	6	T_3+4_/T_1+2_	409/230	0.88 (0.42–1.82)	0.73	68.8	0.01
Clinical stage	9	III + IV/I+II	307/288	2.56 (1.33–4.92)	0.01	55.9	0.02
Lymph node metastasis	8	Yes/no	379/358	1.69 (0.76–3.75)	0.2	77.4	< 0.01
Diameter	4	> 5 cm/< 5 cm	100/98	1.37 (0.74–2.52)	0.32	0	0.91
Location	7	Up and middle/down	456/237	0.79 (0.54–1.14)	0.2	0	0.6

No., number of studies.

### The accuracy of testing for *CDKN2A* methylation for EC diagnosis

**Figure 5:**
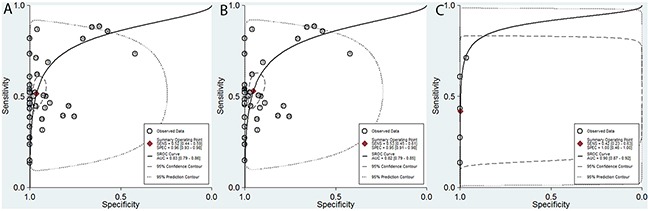
Summary of receiver operating characteristic (SROC) plots of methylated *CDKN2A* for the diagnosis of esophageal cancer based on all samples, tissue samples, and blood samples **(A)** All samples: sensitivity, 0.52 (95% CI, 0.44–0.59); specificity, 0.96 (95% CI, 0.93–0.98); AUC, 0.83 (95% CI, 0.79–0.86). **(B)** Tissue samples: sensitivity, 0.53 (95% CI, 0.45–0.61); specificity, 0.95 (95% CI, 0.91–0.98); AUC, 0.82 (95% CI, 0.79–0.85). **(C)** Blood samples: sensitivity, 0.42 (95% CI, 0.23–0.63); specificity, 1.00 (95% CI, 0.46–1.00); AUC, 0.90 (95% CI, 0.87–0.92).

**Figure 6:**
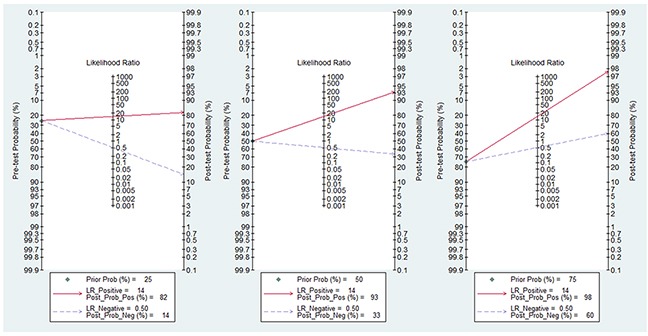
Fagan plot analysis to evaluate the clinical applicability of screening for methylated *CDKN2A* in esophageal cancer diagnosis **(A)** The post-test probability was 82% at a pretest probability of 25%. **(B)** The post-test probability was 93% at a pretest probability of 50%. **(C)** The post-test probability was 98% at a pretest probability of 75%.

In the current analysis, the diagnostic value of methylated *CDKN2A* for EC diagnosis was assessed using data from 41 eligible case-control studies. The pooled sensitivity and specificity values were 0.52 (95% CI, 0.44–0.59) and 0.96 (95% CI, 0.93–0.98), respectively. The area under the summary receiver operating characteristic (SROC) curve (AUC) based on the specificity and sensitivity was 0.83 (95% CI, 0.79–0.86) (Figure 5A). The AUCs based on subgroups where methylation was tested in tissue and blood samples were 0.82 (95% CI, 0.79–0.85) (Figure 5B) and 0.90 (95% CI, 0.87–0.92) (Figure 5C), respectively. As shown in the Figure 6, Fagan plot analysis demonstrated that the probabilities of a patient being diagnosed with EC were 82%, 93%, and 98% following a positive result for methylation of *CDKN2A*, where the pretest probabilities of being diagnosed with EC were set to 25%, 50%, and 75%, respectively; when the test was negative, the probabilities of diagnosis with EC reduced to 14%, 33%, and 60%, respectively.

## DISCUSSION

EC is a complex, progressive disease with multiple stages. Barrett's esophagus (BE), a metaplastic condition where the squamous-lined esophageal mucosa is replaced by specialized intestinal mucosa, and dysplasia, are precursor lesions of EC [2, 22].

Epigenetic modifications, including DNA methylation, contribute significantly to the pathogenesis of EC. DNA methylation is a relatively early molecular change that is a candidate biomarker in several carcinomas [23]. Increasing evidence demonstrates that methylation of the promoter of *CDKN2A*, an important negative regulator of cell growth and proliferation [24], is involved in the tumorigenesis and progression of various tumors, including pancreatic [25], gastric [26], and prostate [27] cancers. Although there have been several reports that the frequency of *CDKN2A* promoter methylation is higher in EC samples than those from cancer-free controls, the association and the role of *CDKN2A* promoter methylation in EC remain controversial. We performed this meta-analysis to address these issues, achieve insights into the role of *CDKN2A* promoter methylation in EC carcinogenesis, and evaluate its diagnostic value in screening for early stage EC.

The current study incorporated a total of 41 case-control studies, including 2487 ECs, 618 precancerosis (including dysplasia and BE), and 2367 control samples. Our results indicate that the frequency of methylation of the *CDKN2A* promoter was significantly higher in EC than in healthy controls and precancerous lesions. Moreover, the level of *CDKN2A* methylation was also remarkably higher in precancerous lesions than in healthy controls. This evidence supports the association of methylated *CDKN2A* with EC carcinogenesis. Recent research has revealed that ESCC and EAC have different molecular features [28]. In our results, the OR of the association with *CDKN2A* methylation for the EAC subgroup was greater than that for the ESCC subgroup, indicating that this molecular feature may be more relevant in EAC. The subgroup analysis by ethnicity showed that *CDKN2A* promoter methylation was associated with an increased risk of EC in Asian and Caucasian, as well as African population, with the Caucasian population showing a higher OR than the Asian and African population, suggesting that the Caucasian population may be more susceptible to *CDKN2A* promoter methylation. The subgroup analysis of sample source presented a significant correlation between hypermethylated *CDKN2A* and EC in both tissue and blood samples, and the pooled OR of blood samples was remarkably higher than that of tissue samples, which suggested that hypermethylated *CDKN2A* may be a useful noninvasive biomarker for blood detection.

Additionally, we evaluated the relationship between *CDKN2A* methylation and the clinicopathological characteristics of EC. A previous systematic review reported that there was a trend towards increased frequency of *CDKN2A* methylation according to EC differentiation grade; however, the association was not statistically significant [29]. In contrast, our results indicate that methylation of the *CDKN2A* promoter is significantly more common among patients with poorly differentiated tumors and advanced clinical stage, compared with those with well/moderately differentiated tumors and early stage disease. Together, these results support the hypothesis that hypermethylation of the *CDKN2A* promoter is involved in the progression of EC.

We further assessed the diagnostic potential of *CDKN2A* promoter methylation for EC, based on data from 41 eligible case-control studies. The pooled sensitivity and specificity for all included studies were 0.52 and 0.96, respectively, indicating that *CDKN2A* promoter methylation could be a useful biomarker for diagnosis of EC. In the current study, we also analyzed SROC and AUC statistics. SROC is a comprehensive index synthesizing sensitivity and specificity, which can be used to evaluate the accuracy of diagnostic tests. The SROC curve and AUC can also be used to evaluate diagnostic power, where an AUC closer to 1.0 signifies that the test has better discrimination. The AUC calculated from all studies included in the current analysis was 0.83, indicating that *CDKN2A* promoter methylation is an extremely useful biomarker for EC diagnosis. In addition, there is increasing evidence that abnormal DNA methylation in body fluid samples is a promising biomarker for cancer screening and diagnosis [30, 31]. As a relatively noninvasive test, blood sample collection is generally acceptable to patients and is a promising route for clinical application of methylation screening. Previous studies have reported that abnormal DNA methylation markers in blood samples can be used to diagnose various cancers [32, 33]. Our subgroup analysis showed that the AUC value for the blood sample group was 0.9, higher than that for the tissue group (0.82), which are in accordance with numerous previous studies [34]. The existence of cell-free circulating tumor DNA (ctDNA) in blood sample could be responsible for this phenomenon. This form of circulating DNA is presumably shed from tumors, either through necrosis or apoptosis [35]. It has been reported that tumor-derived somatic alterations in DNA can be detected in ctDNA with broad clinical application, high sensitivity, and specificity [36–38]. All these above indicated that testing for *CDKN2A* methylation in blood samples has potential as a noninvasive tool for the diagnosis of EC. Future rigorous clinical research studies with larger sample sizes will be essential to validate our findings.

The clinical utility of *CDKN2A* methylation was also evaluated by Fagan plot analysis [39, 40]. The results demonstrated that, when the pre-test probabilities were assumed to be 25%, 50%, and 75%, a corresponding 82%, 93%, and 98% of patients would be correctly diagnosed with EC following positive *CDKN2A* methylation tests; moreover, a diagnosis of EC could be ruled out for 86%, 67%, and 40% of patients following negative results. These analyses suggest that methylated *CDKN2A* has good diagnostic power to discriminate patients with EC from healthy individuals.

The present meta-analysis had several limitations. First, only articles published in English and Chinese were included in the study. Second, significant heterogeneity was observed in the current analysis, and its source was not definitively identified. Third, we did not specifically investigate the association of *CDKN2A* methylation and different EC histology subtypes; therefore, an updated meta-analysis, including more rigorous studies with large sample sizes in the future would support, and could add to the findings of the present study.

In conclusion, our findings provide strong evidence that *CDKN2A* methylation is involved in the carcinogenesis and progression of EC and that it is a promising biomarker for the diagnosis of EC, especially by screening of blood samples. Future large-scale studies are necessary to support or add to our findings, especially regarding the role of *CDKN2A* methylation in the prediction of prognosis, and to support the clinical application of our findings for the benefit of patients with EC.

## MATERIALS AND METHODS

### Literature search

Five electronic databases (including PubMed, Google Scholar, Web of Science, Embase, and China National Knowledge Infrastructure) were searched for eligible studies until October 20, 2016. The following search terms, and various combinations of them, were used: “*p16*,” “*p16^INK4a^*,” “*p14^arf^”, “p14”*, “*CDKN2A (p16 and p14)*,” “cyclin-dependent kinase inhibitor 2A,” “methylation,” “DNA methylation,” “promoter methylation,” “esophageal carcinoma,” “esophagus cancer,” “esophageal tumor,” and “esophageal malignancy.” In addition, we performed a manual search of references to find potentially relevant articles.

### Selection criteria

We collected all eligible articles that addressed the association between *CDKN2A* methylation and EC. Articles with data meeting the following criteria were included: (1) all samples confirmed by pathology, including ECs, esophageal precancerous lesions (dysplasia or Barrett's esophagus), and healthy controls; (2) case-control or cohort studies of *CDKN2A* promoter methylation in EC; (3) included sufficient data regarding the methylation frequency of the *CDKN2A* promoter to enable the calculation of odds ratios and 95% confidence intervals; and (4) where authors published several reports using the same population or overlapping data, only the most complete study with the most information was selected.

### Data quality assessment

The quality of studies was assessed according to Newcastle–Ottawa Scale (NOS) criteria [41]. The NOS evaluation system includes three aspects: (1) subject selection: 0–4 points; (2) comparability of subjects: 0–2 points; and (3) clinical outcome: 0–3 points. NOS scores range from 0 to 9; and a score ≥ 7 indicates good quality. Only studies with scores ≥ 7 were included in the analysis.

### Data extraction

Three authors (QL, JL, and CZ) independently reviewed all the available articles and extracted relevant data from eligible articles. The following information was recorded: the first author's name; the country, ethnicity of subjects, year of publication, sample type, number of cases, control type, and detection method of methylation of the study; and the methylation frequency of *CDKN2A*, and clinicopathological parameters (including age, gender, smoking behavior, alcohol consumption, tumor location, tumor diameter, differentiation grade, tumor stage, clinical stage and lymph node metastasis) of the subjects. Inconsistent information was discussed by all authors and a consensus reached on its use.

### Statistical analyses

Data were analyzed using Stata statistical software, version 12.0 (Stata Corporation, College Station, TX, USA). Overall odds ORs and corresponding 95% CIs were calculated to evaluate the strengths of the associations between *CDKN2A* methylation and different EC pathological processes (cancer vs. healthy control, cancer vs. precancerous lesion, precancerous lesion vs. healthy control), as well as clinicopathological features. Potential heterogeneity was quantified using Cochran's Q statistic and *I^2^* tests [42]. When significant heterogeneity was observed among studies, a random-effects model (DerSimonian-Laird method) [43] was applied to calculate a pooled OR (*I^2^* > 50% or *P* < 0.05), otherwise, a fixed-effects model (Mantel–Haenszel method) was used [44]. Meta-regression and subgroup analyses were performed to explore the sources of heterogeneity. Sensitivity analyses were conducted to determine the influence of individual studies on the pooled results, through the omission of single studies [45]. Publication bias was quantitatively assessed using Begg's linear regression tests [46]. Pooled sensitivity and specificity were used to estimate the diagnostic efficacy of testing for *CDKN2A* promoter methylation. Two indicators, the SROC and AUC, were also calculated to evaluate the stability and accuracy of the diagnostic capacity of *CDKN2A* promoter methylation for EC [47]. Fagan plot analysis was performed with 25%, 50%, and 75% pre-test probability to assess the diagnostic power of *CDKN2A* promoter methylation in clinical practice [39]. All *P* values are two-sided and *P* values < 0.05 were considered statistically significant.

## Abbreviations

EC: esophageal cancer
EAC: esophageal adenocarcinoma
ESCC: esophageal squamous cell carcinoma
BE: Barrett's esophagus
*CDKN2A*: cyclin-dependent kinase inhibitor 2A
SROC: summary receiver operating characteristic
AUC: the area under the SROC
NOS: the Newcastle–Ottawa Scale
OR: odds ratio
CI: confidence interval
